# *ciaR* impacts biofilm formation by regulating an arginine biosynthesis pathway in *Streptococcus sanguinis* SK36

**DOI:** 10.1038/s41598-017-17383-1

**Published:** 2017-12-07

**Authors:** Bin Zhu, Xiuchun Ge, Victoria Stone, Xiangzhen Kong, Fadi El-Rami, Yan Liu, Todd Kitten, Ping Xu

**Affiliations:** 1 Philips Institute for Oral Health Research, Virginia Commonwealth University, Richmond, VA 23298 United States of America; 2 College of Biochemical Engineering, Anhui Polytechnic University, 241000 Wuhu, China; 3 Microbiology and Immunology Department, Virginia Commonwealth University, Richmond, VA 23298 United States of America

## Abstract

*Streptococcus sanguinis* is an early colonizer of the tooth surface and competes with oral pathogens such as *Streptococcus mutans* to maintain oral health. However, little is known about its mechanism of biofilm formation. Here, we show that mutation of the *ciaR* gene, encoding the response regulator of the CiaRH two-component system in *S*. *sanguinis* SK36, produced a fragile biofilm. Cell aggregation, *gtfP* gene expression and water-insoluble glucan production were all reduced, which suggested polysaccharide production was decreased in Δ*ciaR*. RNA sequencing and qRT-PCR revealed that arginine biosynthesis genes (*argR*, *argB*, *argC*, *argG*, *argH* and *argJ*) and two arginine/histidine permease genes (SSA_1568 and SSA_1569) were upregulated in Δ*ciaR*. In contrast to Δ*ciaR*, most of strains constructed to contain deletions in each of these genes produced more biofilm and water-insoluble glucan than SK36. A Δ*ciaR*Δ*argB* double mutant was completely restored for the *gtfP* gene expression, glucan production and biofilm formation ability that was lost in Δ*ciaR*, indicating that *argB* was essential for *ciaR* to regulate biofilm formation. We conclude that by promoting the expression of arginine biosynthetic genes, especially *argB* gene, the *ciaR* mutation reduced polysaccharide production, resulting in the formation of a fragile biofilm in *Streptococcus sanguinis*.

## Introduction

Biofilms are microbial communities embedded in a self-produced matrix of extracellular polymeric substances of bacterial origin^1^. They are variously composed of polysaccharides, proteins, nucleic acids and lipids, which mediate cell adhesion to solid surfaces and form cohesive, three-dimensional polymer networks^2^. Clinically, biofilms are a significant risk factor in medical-device related infections and are highly associated with chronic infections, such as infective endocarditis, periodontal disease and cystic fibrosis^3,4^.

In the oral cavity, biofilm in the form of dental plaque is a highly organized, multi-species network initiated by the colonization of oral streptococci. *Streptococcus sanguinis*, an indigenous gram-positive bacterium, has long been recognized as a pioneering colonizer, aiding in the attachment of succeeding organisms, and a key player in plaque biofilm development^5,6^. Several studies have examined the importance of *S*. *sanguinis* in early colonization^7,8^. It has been demonstrated that *S*. *sanguinis* can compete with pathogenic bacterial species associated with oral diseases. One of the most well-studied examples is the antagonism between *S*. *sanguinis* and *Streptococcus mutans*, a predominant contributor to dental caries formation^7,9–14^. However, there are only a few studies focusing on the mechanism of biofilm formation by *S*. *sanguinis* ^15^.

Arginine is reported to be detrimental for biofilm formation in *S*. *mutans* ^16–18^. Treatment with 15 mg/mL of L-arginine (a clinically effective concentration) decreased the proportion of S. *mutans*, increased the proportion of *Streptococcus gordonii*, and maintained *Actinomyces naeslundii* proportion within biofilms^17^. Moreover, L-arginine treatment reduced the amount of insoluble extracellular polysaccharide production^17,18^, which significantly altered the architecture of the biofilm in *S*. *mutans* ^17^. In addition, metabolism of arginine by bacteria possessing the arginine deiminase system results in increased pH, which protects against caries caused by *S*. *mutans* and other aciduric bacteria^19,20^.

The two-component regulatory system (TCS) CiaRH has been shown to affect β-lactam resistance, cell lysis, genetic competence and virulence in *Streptococcus pneumoniae* ^21–24^. In *S*. *mutans*, CiaRH controls bacteriocin production, genetic competence, and tolerance to environmental stresses^25–27^. In addition, the biofilm formation of a *ciaH* mutant was defective in *S*. *mutans*, but the reason for this effect was unclear^27^.

CiaR is a response regulator of the CiaHR two-component system. For *S*. *pneumoniae* and *S*. *mutans*, activated *CiaR* acts as a repressor of DNA uptake by reducing the concentration of the competence stimulating peptide (CSP), a small (16 to 19-residue) unmodified peptide pheromone^28–30^. *CiaR* represses *comC* (the gene responsible for CSP biosynthesis) at the transcriptional level through single csRNAs in *S*. *pneumoniae* ^29^ and promotes the transcription of *htrA*, whose protein product directly digests CSP^24,31^. The genetic competence system can also influence biofilm formation as CSP-induced cell death was shown to contribute to the release of chromosomal DNA into the extracellular matrix of *S*. *mutans* biofilms^32–34^.

Amino acid alignments suggest that SSA_0959 encodes CiaR and SSA_0960 encodes CiaH in *S*. *sanguinis* SK36. Secondary structures of CiaH and CiaR were predicted by SMART (http://smart.embl.de/)^35^. CiaH has two transmembrane domains, a phosphoacceptor domain, and a histidine kinase-like ATPase domain, which presumably senses a stimulus and transfers a signal to the response regulator (Fig. S1). CiaR contains a CheY-homologous receiver domain and a transcriptional regulatory domain, which likely receives the signal and mediates the cellular response (Fig. S1).

In this study, we showed that mutation of *ciaR* in *S*. *sanguinis* SK36 resulted in a fragile biofilm. By testing the concentration of water-insoluble glucan (WIG), the expression of the *gtfP* gene and the binding ability of polysaccharide-specific fluorescent dye, we confirmed that polysaccharide production was decreased in the Δ*ciaR* mutant, which resulted in biofilm formation deficiency. These phenotypes of Δ*ciaR* could be restored by Δ*ciaR*Δ*argB* double mutation, indicating that *argB* was essential for *ciaR* to regulate biofilm formation. We conclude that this decrease in GtfP-mediated polysaccharide production was responsible for the observed deficiency of this mutant for biofilm formation.

## Results

### Mutation of *ciaR* decreases biofilm formation in *S*. *sanguinis* SK36

**Figure 1:**
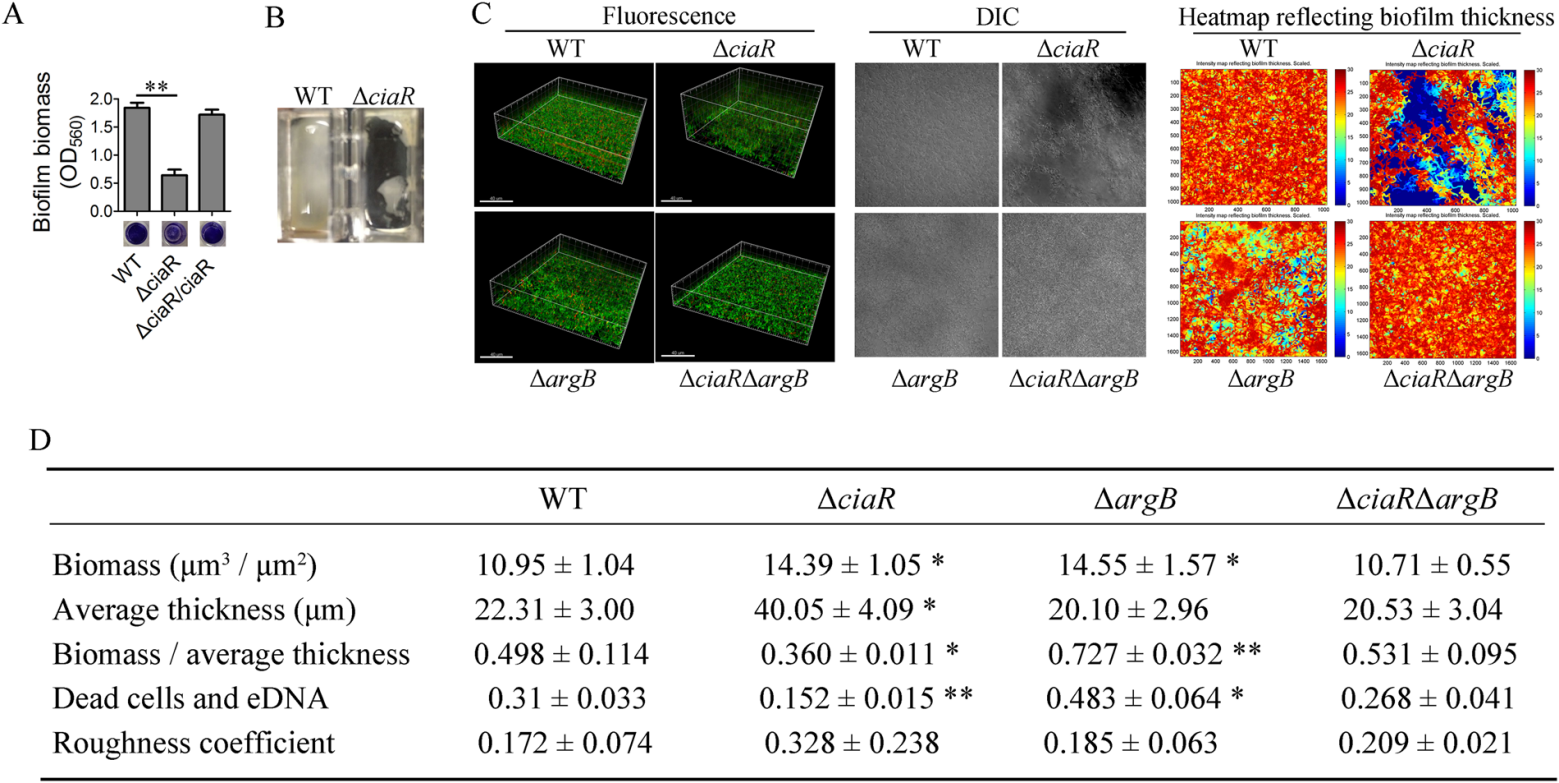
The impact of *ciaR* mutation on biofilm formation. (**A**) The biofilms of WT, Δ*ciaR* and Δ*ciaR*/*ciaR* were cultured in a 96-well plate in BM media for 24 hours. Biomass was measured by crystal violet staining. (**B**) Biofilms of WT and Δ*ciaR* cultured in 4-well chambers after being washed with PBS buffer. (**C**) Biofilms grown in 4-well chambers were stained by SYTO9 and propidium iodide. Fluorescence (left) and differential interference microscopy images (middle) were obtained by confocal laser scanning microscopy. Fluorescence images were analyzed by COMSTAT script, and heat maps of biofilm thickness were generated, which showed the distribution of biomass in biofilms (right). (**D**) The fluorescence images were analyzed by COMSTAT. Biofilm biomass, average thickness, propidium iodide signal and roughness coefficient were quantified, respectively. All the data in Fig. 1D were compared with their WT control. *P ≤ 0.05, **P ≤ 0.01, Student’s *t-*test. Means and standard deviations from triplicate experiments are shown.

Because *S*. *sanguinis* is a pioneering colonizer and a key player in plaque biofilm development^5–7^, we were interested in examining the mechanism of biofilm formation in *S*. *sanguinis*. In our previous work, a comprehensive mutant library of *S*. *sanguinis* SK36 was generated by high-throughput PCR^36,37^. By using a microtiter dish biofilm assay, we tested the biofilm formation ability of all mutants predicted to be two-component system regulators. Our initial screening indicated that deletion of the *ciaR* gene (SSA_0959) resulted in a decreased biofilm phenotype compared to the wild-type SK36 (Fig. 1A). This phenotype was restored in the Δ*ciaR*-complemented strain, Δ*ciaR*/*ciaR* (Fig. 1A). The wild-type strain (WT) and Δ*ciaR* were statically cultured in biofilm media (BM) for 24 hours in a 4-well chamber then stained by SYTO9/propidium iodide (PI), which marked live/dead cells, respectively. This resulted in Δ*ciaR* forming a loose biofilm, which was easily broken (Fig. 1B). These biofilms were subsequently observed by confocal laser scanning microscopy (CLSM) and quantified using a COMSTAT script in Matlab software^38^. Although containing more biomass by this analysis, the biofilm of Δ*ciaR* was also much thicker and as a result had a lower ratio of biomass/average thickness (Fig. 1C and D). In other words, the cell density inside the Δ*ciaR* biofilm was less than that of WT. The differential interference contrast (DIC) images and the roughness coefficient illustrated that the biofilm of Δ*ciaR* had an irregular structure (Fig. 1C and D). Heat maps of biofilm thickness were generated using the COMSTAT script, which showed the distribution of biomass in biofilms. The biomass of WT was uniformly distributed, but there were many large gaps within the Δ*ciaR* biofilm (Fig. 1C). These results indicate that the biofilm structure was impacted by *ciaR* mutation.

**Figure 2:**
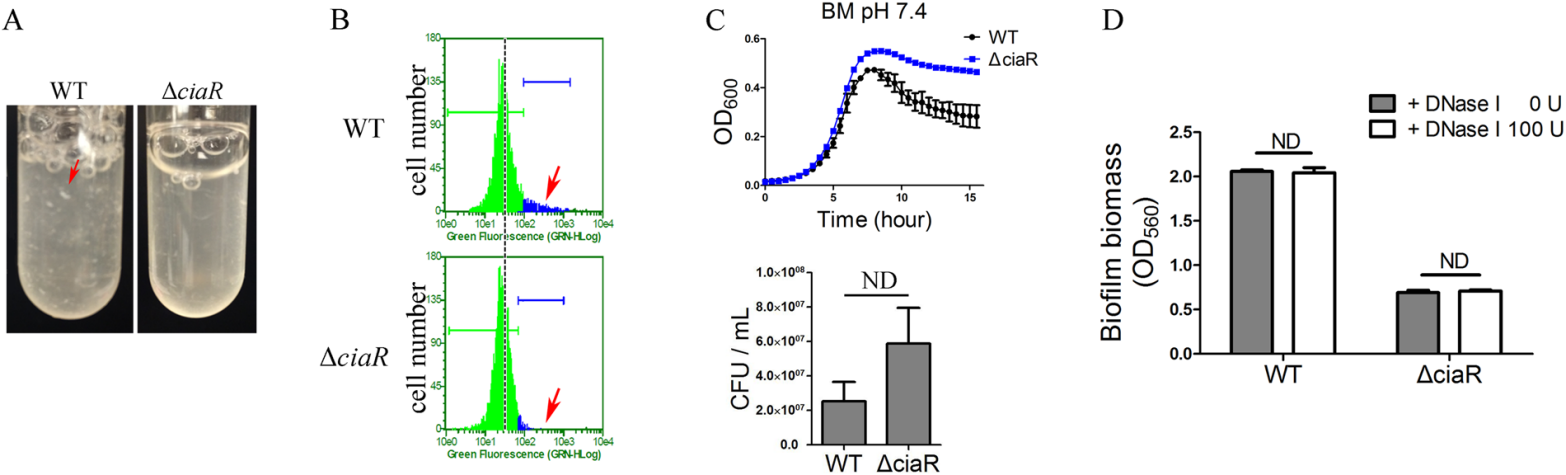
Polysaccharide production and cell growth of WT and Δ*ciaR*. (**A**) Strains were incubated in BM at a shaking speed of 200 rpm for 24 hours. The arrow points a cellular auto-aggregate. (**B**) Strains were incubated in BM at a shaking speed of 200 rpm for 24 hours. Extracellular polysaccharide was stained by HHA-FITC and measured by flow cytometry. Arrow indicates a tail of increased staining in the WT strain. (**C**) WT and Δ*ciaR* were cultured in BM. Every 30 minutes, cells were shaken for 3 minutes and OD_600_ was read using a platereader (Top). CFU values were determined after 16 hours of incubation (bottom). (**D**) Biofilms were cultured in BM for 24 hours and then treated with 100 U/mL of DNase I for 1 hour. Microtiter dish biofilm assay was performed after DNase I treatment. Means and standard deviations from triplicate experiments are shown. ND: no significant difference, Student’s *t-*test.

Extracellular polysaccharide is one of the most important components of many biofilm matrixes^2,39^. The phenotype of cell auto-aggregation has been linked with over-production of extracellular polysaccharide in other bacterial species^40,41^. When WT and Δ*ciaR* strains were incubated in BM at 200 rpm for 24 hours, cell aggregation was observed only in the WT culture, suggesting that polysaccharide concentration might be reduced in Δ*ciaR* (Fig. 2A). The extracellular polysaccharide of WT and Δ*ciaR* was stained by *Hippeastrum* hybrid lectin (HHA)-FITC (EY Labs, USA) and measured by flow cytometry^42^. Compared with Δ*ciaR*, a subpopulation of WT cells had increased staining and, presumably, a higher concentration of polysaccharide, which might promote auto-aggregation and biofilm formation (Fig. 2B).

Growth curves of WT and Δ*ciaR* were generated in BM with a microtiter plate reader. Δ*ciaR* had a higher value of OD_600_ and no significant change of colony forming units (CFU) (Fig. 2C). Because cell aggregation might impact OD_600_, the growth curves and CFU were also measured in BHI with continuous shaking. Similar to the result in BM medium, the final OD_600_ values of Δ*ciaR* were higher than WT in BHI (Fig. S2). Cell cultures were sonicated to reduce auto-aggregation and then CFUs were determined. The CFUs from the Δ*ciaR* culture were even higher than that of WT. Taken together, the results suggest that the deficiency of biofilm formation seen in Δ*ciaR* was not caused by impaired cell growth (Fig. S2).

Extracellular DNA (eDNA) is also an essential component of the biofilm matrix in many species^2,39^. The amount of dead cells and eDNA in Δ*ciaR* was less than that of WT, suggesting that *ciaR* might impact biofilm formation by reducing eDNA levels. To examine this possibility, the biofilms of WT and Δ*ciaR* were cultured in BM for 24 hours and then treated with DNase I (100 U/mL, QIAGEN) for 1 hour. The biofilm assay was repeated. The addition of DNase I did not affect the biofilm biomass of WT or Δ*ciaR* (Fig. 2D). The same result was observed if DNase I was added at the beginning (not shown). These results suggest that the *ciaR* mutation did not reduce biofilm formation by decreasing eDNA production.

Taken together, the data suggest that Δ*ciaR* may impact biofilm formation by inhibiting polysaccharide production. To further address the mechanism, RNA sequencing was done to explore the change of gene expression in Δ*ciaR*.

### Δ*ciaR* activates the arginine biosynthesis pathway

**Figure 3:**
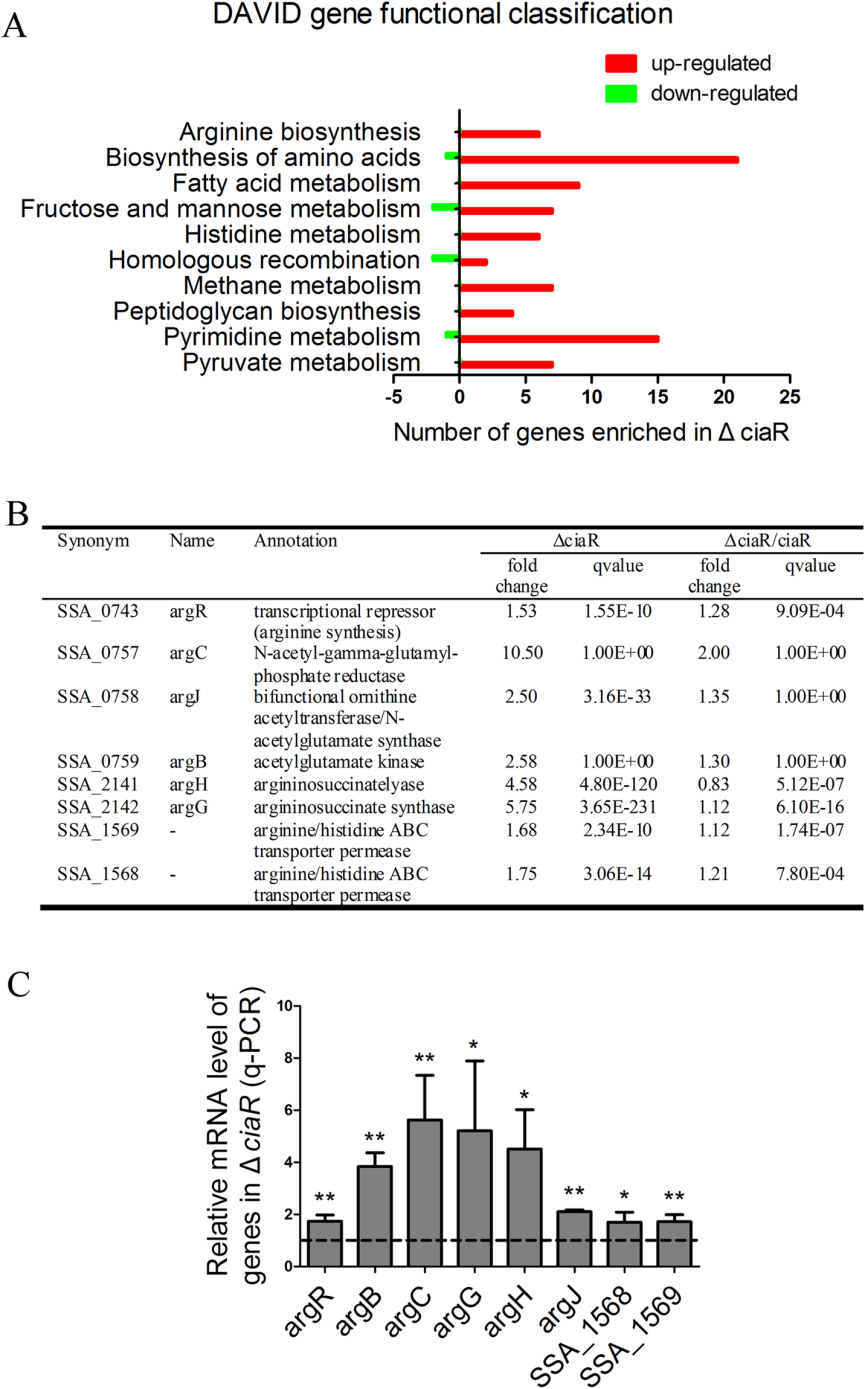
Differentially expressed genes in Δ*ciaR*. (**A**) Genes with fold change ≥ 1.5 or ≤ 0.67 and qvalue ≤ 0.01 in Δ*ciaR* RNA sequencing data were analyzed by DAVID gene functional classification tools. The enriched pathways were shown. (**B**) Transcript levels of *arg* genes in Δ*ciaR* and Δ*ciaR*/*ciaR*. (**C**) qRT-PCR was performed to examine the expression of *arg* genes in Δ*ciaR*. Means and standard deviations from triplicate experiments are shown.*P ≤ 0.05, **P ≤ 0.01, Student’s *t-*test.

RNA sequencing data were generated and analyzed (Supplementary Dataset). There were 309 genes up-regulated (fold change ≥ 1.5, qvalue ≤ 0.01) and 190 genes down-regulated (fold change ≤ 0.667, qvalue ≤ 0.01). These regulated genes were analyzed by DAVID gene functional classification tools to identify enriched functional groups^43,44^ (Fig. 3A). Mutants deleted for the enriched differentially expressed genes were selected and examined for their biofilm formation using our comprehensive gene deletion mutant library and a high-throughput biofilm assay. Based on the biofilm deficiency in the *ciaR* mutant, if a gene was up-regulated in Δ*ciaR* and the mutant deleted for this gene had more biofilm, this gene was judged as positively related to Δ*ciaR* biofilm formation. If a gene was down-regulated in Δ*ciaR* and the mutant deleted for this gene had more biofilm, it was negatively related. Most of the pathways enriched in DAVID analysis contained only a few genes that were positively related to Δ*ciaR* biofilm formation. The exception was the arginine biosynthesis pathway.

We found that all of the *arg* genes associated with arginine biosynthesis were up-regulated in Δ*ciaR* (Fig. 3B) and biofilm formation by all but one of these arginine biosynthesis-related mutants was increased (Fig. S3), which led us to hypothesize that *ciaR* mutation inhibited biofilm formation by activating arginine biosynthesis. qRT-PCR confirmed that the expression of arginine biosynthesis genes (*argR*, *argB*, *argC*, *argG*, *argH* and *argJ*) and two arginine/histidine permease genes (SSA_1568 and SSA_1569) was significantly increased in Δ*ciaR* (Fig. 3C).

### Δ*ciaR* reduces biofilm formation by activating the arginine biosynthesis pathway

To further test the hypothesis, eight double mutants (Δ*ciaR* combined with Δ*argR*, Δ*argB*, Δ*argC*, Δ*argG*, Δ*argH*, Δ*argJ*, ΔSSA_1568 and ΔSSA_1569) were constructed. Firstly, the growth of these strains, along with that of the parent strains, was examined in BHI with continuous shaking. The growth rates and maximum optical densities of Δ*argR*, Δ*argC*, Δ*argG*, Δ*argH*, Δ*argJ*, ΔSSA_1568 and ΔSSA_1569 were all less than WT (Fig. S4A–G). It has been reported that alkali generation through ammonia production, especially from arginine, is essential for maintaining pH homeostasis in the oral cavity^20^. The deletion of arginine biosynthesis genes might decrease arginine production and as a result affect cell growth. The OD_600_ of Δ*argR*, Δ*argC*, Δ*argG*, Δ*argH*, ΔSSA_1568 and ΔSSA_1569 was less than or similar to that of Δ*ciaR* (Fig. S4A–D and F–G). These results suggested that the increased biofilm levels observed in these *arg* mutants was not caused by a change in cell growth. The Δ*argB* mutant exhibited severe auto-aggregation even when cultured in BHI medium with continuous shaking (Fig. S4I).

**Figure 4:**
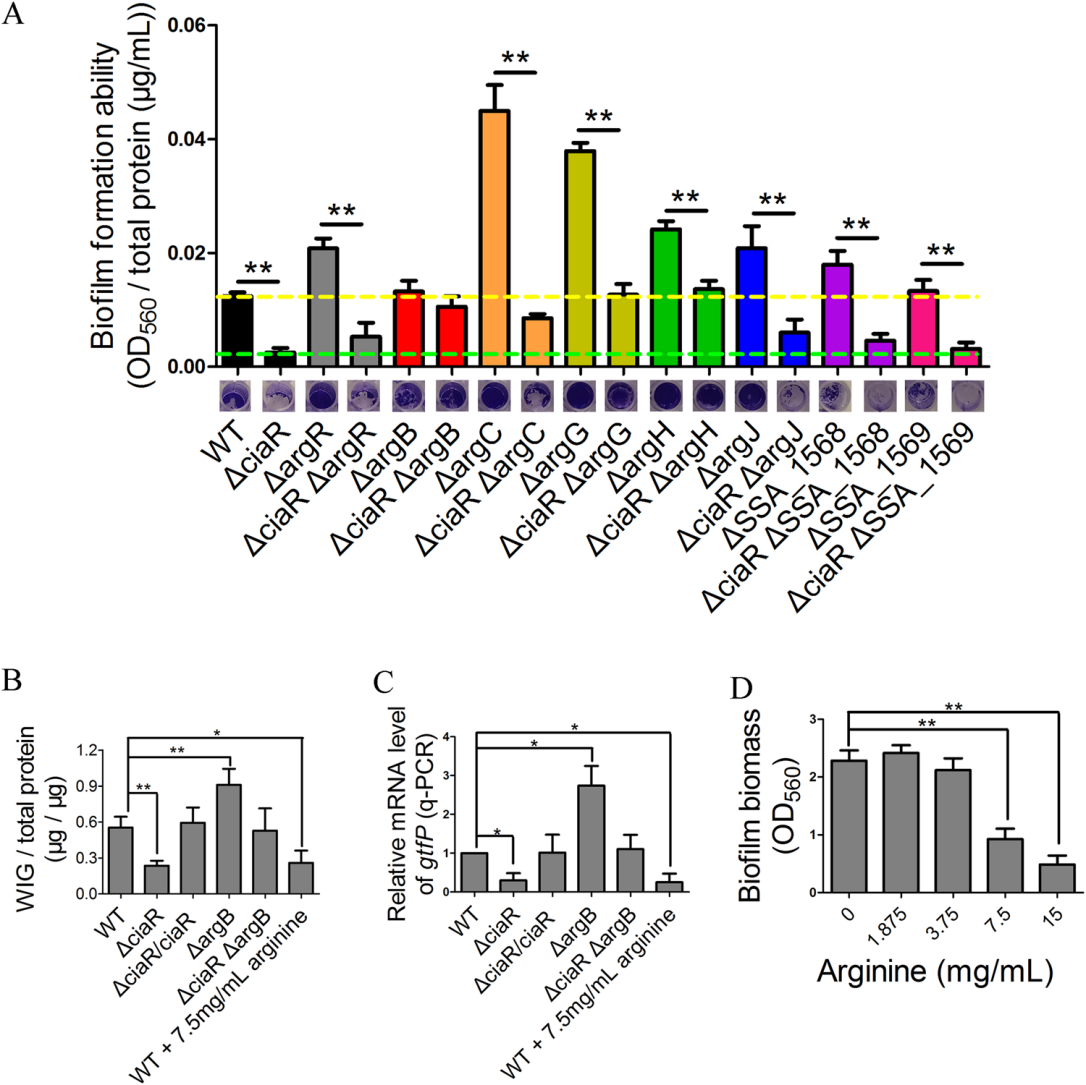
Δci*aR* impacts biofilm formation by modulating the expression of arginine biosynthesis genes. (**A**) Biofilm biomass was examined by microtiter dish biofilm assay and at the same time, the total protein concentration was quantified by BCA assay. The biofilm formation ability was defined as biomass (OD_560_) divided by total protein concentration (μg/mL). (**B**) Biofilms of strains were grown in BM for 24 hours in 24-well plates. WIG levels were assayed. (**C**) qRT-PCR assay was performed to show the expression of *gtfP* gene in different strains or in WT cultured in BHI with different concentrations of L-arginine. (**D**) WT was cultured in BM with different concentrations of L-arginine. Biofilm formation ability was examined by microtiter dish biofilm assay. Means and standard deviations from triplicate experiments are shown. *P ≤ 0.05, **P ≤ 0.01, Student’s *t-*test.

To ensure the measurement of biofilm formation was not impacted by differences in growth, we tested the biofilm biomass by a microtiter dish biofilm assay and at the same time quantified the total protein concentration of each sample (including the protein of cells in the supernatant). Biofilm formation was defined as biomass (OD_560_) divided by total protein concentration (μg/mL). There was no significant difference of biofilm formation ability between Δ*argB* and Δ*ciaR*Δ*argB* (Fig. 4A).

The biofilm structures of Δ*argB* and Δ*ciaR*Δ*argB* were measured by CLSM. As mentioned above, the mutation of *ciaR* reduced cell density of biofilm (biomass / average thickness ratio) and resulted in the biofilm with an irregular structure. In contrast, deletion of *argB* significantly increased cell density (Fig. 1D). The cell density and biomass distribution of Δ*ciaR*Δ*argB* were similar to that of WT, indicating that mutation of *argB* could restore the biofilm formation ability of Δ*ciaR* to WT levels (Fig. 1C and D).

We next examined whether the effect of the *ciaR* mutation on polysaccharide production could be related to arginine biosynthesis. The generation of WIG is positively related with the concentration of polysaccharide^15,21^ and controlled by GtfP, the only glucosyltransferase present in *S*. *sanguinis* ^15,45^. In Δ*ciaR*, WIG production was half that of WT (Fig. 4B). This phenotype was restored in the complemented mutant, Δ*ciaR*/*ciaR*. In RNA sequencing experiment, the expression of *gtfP* in Δ*ciaR* was about one-fifth that of WT; however, because the reads of *gtfP* were low, it did not show a significant difference. Quantitative RT-PCR (qRT-PCR) was performed to confirm that *gtfP* was down-regulated in Δ*ciaR* and restored in Δ*ciaR*/*ciaR* (Fig. 4C).

The concentration of WIG in Δ*argB* was increased and the Δ*ciaR*Δ*argB* mutant produced WT levels of WIG (Fig. 4B). Interestingly, the *gtfP* gene was over-expressed in Δ*argB* whereas the Δ*ciaR*Δ*argB* double mutant expressed *gtfP* at WT levels (Fig. 4C). These data are consistent with a model whereby CiaR promotes biofilm formation by suppressing *argB* transcription, which in turn, increases *gtfP* expression and concomitant polysaccharide production.

Compared with WT, Δ*argR*, Δ*argC*, Δ*argG*, Δ*argH*, Δ*argJ* and ΔSSA_1568 produce more biofilm (Fig. 4A). Moreover, Δ*argR*, Δ*argC*, Δ*argG*, ΔSSA_1568 and ΔSSA_1569 produced more WIG (Fig. S5). Mutation of *ciaR* was defined as one factor impacting biofilm formation, and the mutation of *arg* gene was defined as another, then two-way ANOVA was utilized to analyze whether *ciaR* is influenced by *arg* genes for biofilm formation. As shown in Table S1, the p values of column factors were all less than 0.0001, illustrating that the mutation of *ciaR* could affect biofilm formation. The p values of the row factors Δ*argR*, Δ*argB* Δ*argC* Δ*argG* Δ*argH* Δ*argJ* and ΔSSA_1568 were less than 0.05, suggesting these mutations could also modulate biofilm formation. The *argR*, *argB*, *argC* and *argG* mutations significantly interacted with *ciaR* mutation to alter biofilm formation, but Δ*argH*, Δ*argJ*, ΔSSA_1568 and ΔSSA_1569 altered biofilm formation independent from *ciaR*, which suggests that not only *argB*, but also *argR*, *argC* and *argG* are regulated by *ciaR* to modulate biofilm formation.

GtfP utilizes sucrose but not glucose for polysaccharide production^45^. When biofilms were examined in cells cultured in BM + 1% glucose instead of 1% sucrose, *gtfP* mutation had no effect on biofilm formation (Fig. S6). The biofilm biomass of Δ*argR* and Δ*argC* mutants was almost the same as that of WT (Fig. S6). The biomass of the Δ*argB* and Δ*argG* mutants was even lower than WT (Fig. S6). Compared with that in BM + 1% sucrose, the biofilm formation ability of Δ*argR*, Δ*argB*, Δ*argC* and Δ*argG* were all decreased in BM + 1% glucose (Fig. S6). These data also suggested that *arg* genes affect biofilm formation by impacting polysaccharide production.

There have been some reports demonstrating reduction of biofilm formation by addition of arginine^16–18^. We added exogenous arginine to BM medium to test its effect on biofilm biomass using a microtiter dish biofilm assay. Addition of L-arginine to 7.5 mg/mL reduced WIG production and also inhibited the biofilm formation of WT (Fig. 4B and D). Moreover, the expression of *gtfP* in WT was decreased with by the addition of 7.5 mg/mL of L-arginine (Fig. 4C). These data are consistent with the hypothesis that *ciaR* affects biofilm formation by regulating arginine biosynthesis.

L-ornithine is a precursor for L-arginine production^46^. We added different concentrations of L-ornithine to BM, which decreased the biofilm forming ability of WT (Fig. S7). Arc is an ornithine carbamoyltransferase, which is necessary for the conversion of ornithine to arginine^46^. Additional L-ornithine did not impact the biofilm formation of Δ*arc*. These results are also consistent with our conclusion that arginine biosynthesis is inhibitory for biofilm formation.

### *ciaR* affects biofilm formation of *S*. *sanguinis* in flow conditions

Oral biofilms on tooth surfaces are constantly exposed to flow conditions. To model the biofilm formation ability of Δ*ciaR* in the oral cavity, a flow cell system was established. Two fluorescent proteins, mTFP1 (green) and mCherry (red), were expressed from the plasmids pVMTeal and pVMCherry, respectively^47^. Strains expressing mTFP1 or mCherry were cultured in BM with 10 μg/mL erythromycin in the channels of a microfluidics chip with a flow speed of 0.1 μL/min. Images were obtained using fluorescence microscopy at different time points. Four days later, biofilms in the chip were observed by CLSM. Images were analyzed by COMSTAT^38^.

**Figure 5:**
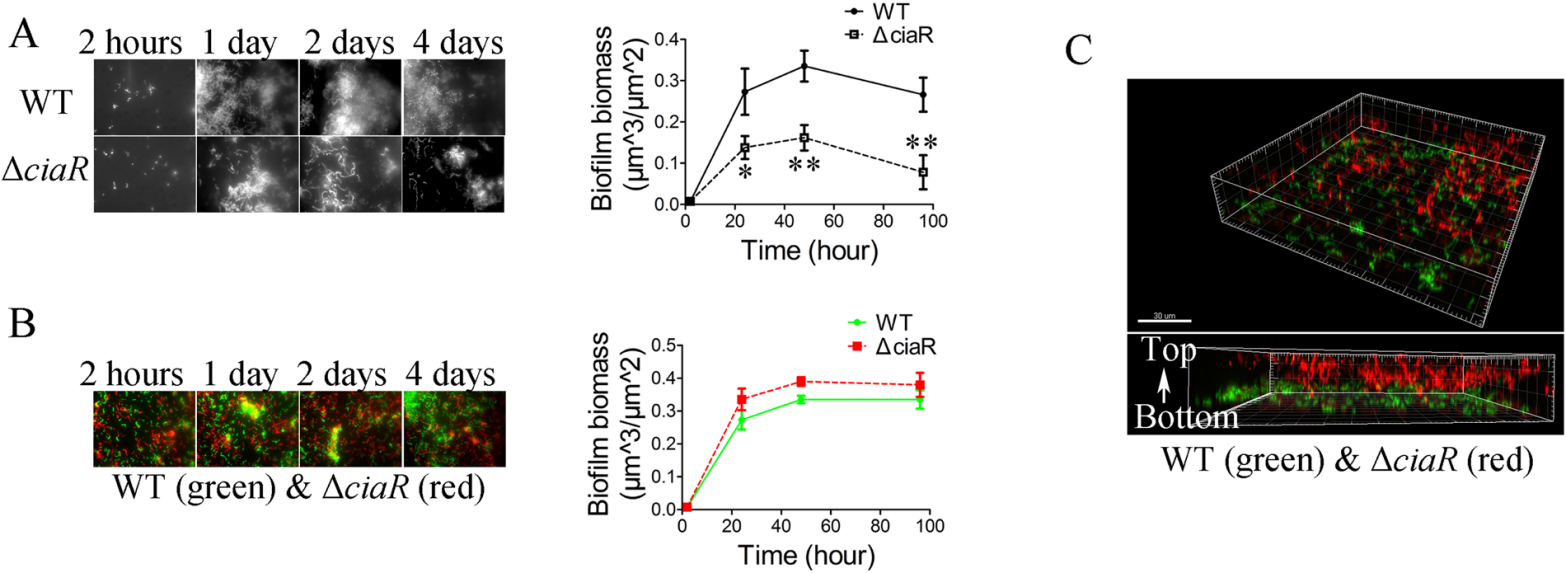
Biofilm formation of WT and Δ*ciaR* under flow cell conditions. Strains were marked by different fluorescent reporters and then cultured in a flow cell system. Image in Fig. 5C was obtained by CLSM. Others were recorded by the fluorescence microscopy. Biofilm biomass was quantified by COMSTAT software. (**A**) The biofilms biomass of WT and Δ*ciaR* in the flow cell system at different time points. (**B**) WT and Δ*ciaR* were co-cultured in the flow cell channel and biofilm biomass was measured. (**C**) After being co-cultured for 4 days, the structure of biofilm formed by WT – Δ*ciaR* was examined by CLSM. For each sample in Fig. 5A and B, ten images from fluorescence microscopy were obtained to calculate the means and standard deviations. *P ≤ 0.05, **P ≤ 0.01, Student’s *t-*test.

Perhaps because Δ*ciaR* cells adhered less well, the biofilm biomass of Δ*ciaR* was much less than that of WT in the flow cell system, which was different from the biofilm biomass tested in the 4-well chamber but similar to the result in the 96-well plate (Fig. 5A). Surprisingly, when Δ*ciaR* was co-cultured with WT, the biomass of Δ*ciaR* was similar to WT (Fig. 5B). CLSM images showed that Δ*ciaR* grew on the surface of the biofilm formed by WT (Fig. 5C). One explanation for this finding is that Δ*ciaR* might be lacking in polysaccharide for attachment and utilize the polysaccharide produced by WT for localization. In this model, reduced polysaccharide production decreases the attachment of Δ*ciaR* in monoculture and as a result, decreases the abundance of Δ*ciaR* in flow conditions.

### Arginine affects biofilm formation in *S*. *sanguinis* by affecting GtfP-mediated polysaccharide production rather than alkali generation

As mentioned above, arginine is utilized by the arginine deiminase system for alkali generation and plays an important role in pH homeostasis in *S*. *sanguinis* ^19,20^. When WT and Δ*ciaR* were cultured overnight in BHI medium, the pH of both strains dropped to 5.56 ± 0.23. In the annotation of the *S*. *sanguinis* SK36 genome, there are two arginine deiminase (*arc*) genes. SSA_0738 (Arc) is an ornithine carbamoyltransferase and SSA_0739 (ArcC) is a carbamate kinase. The expression of these two genes was not changed in Δ*ciaR* (Supplementary Dataset). The tolerance of strains to acid conditions was tested by treatment of cells with an acetic acid - sodium acetate buffer at pH 4.8 for 30 minutes. None of the *arg* mutants exhibited acid sensitivity. In contrast, Δ*argB* survived much better (Fig. S8). It is possible that a higher concentration of polysaccharide or a better ability to auto-aggregate protected Δ*argB* from the acid treatment. It appears that arginine affects biofilm formation in *S*. *sanguinis* by affecting GtfP-mediated polysaccharide production rather than alkali generation.

### *ciaR* is a genetic competence inhibitor in *S*. *sanguinis*

**Figure 6:**
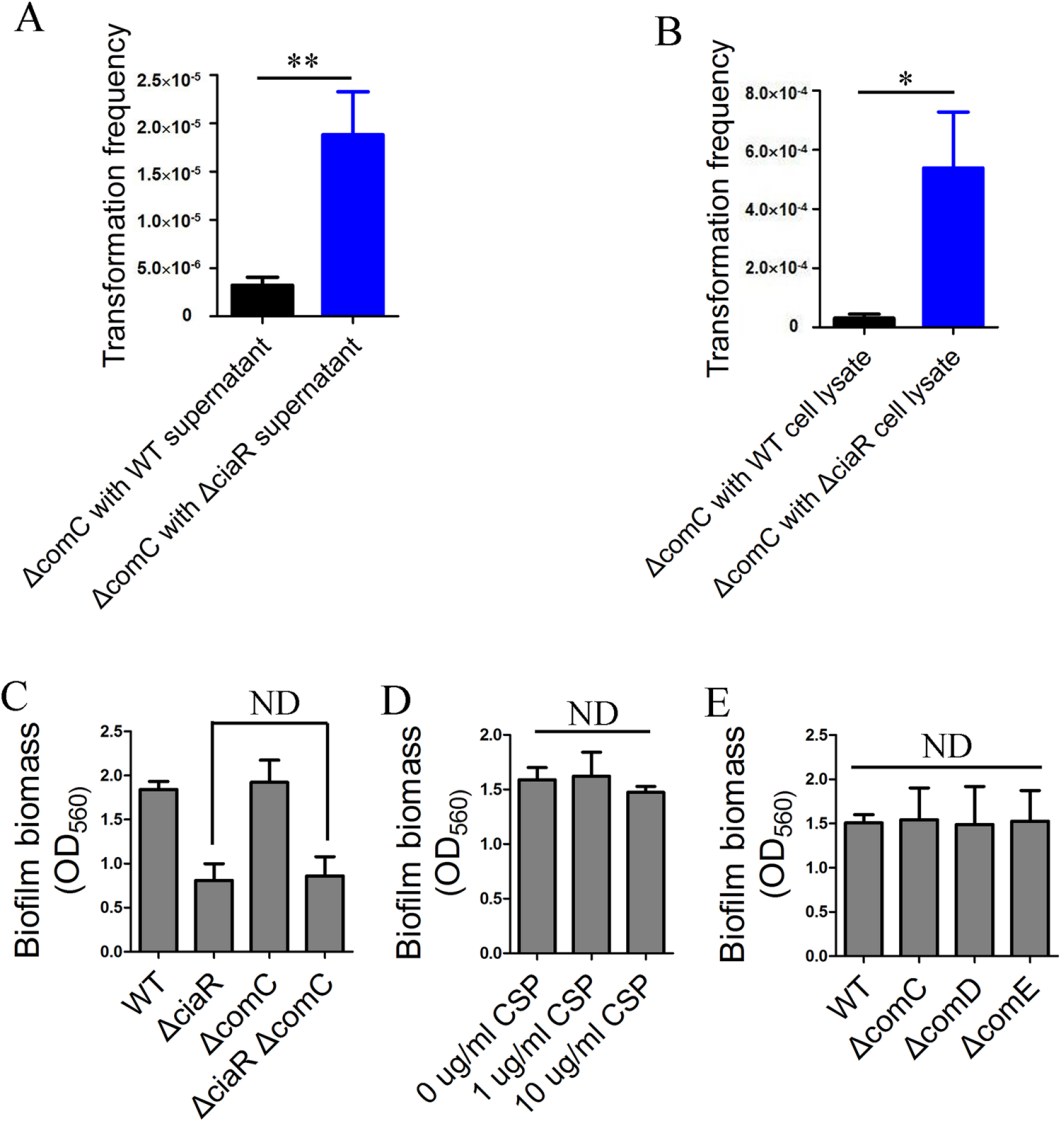
The effect of Δ*ciaR* on competence stimulation and the effect of CSP and competence genes on biofilm formation. The concentration of CSP was quantified by measuring the transformation frequency of a Δ*comC* mutant to which was added culture supernatant (**A**) or cell lysate (**B**) of WT and Δ*ciaR*, respectively. (**C** and **E**) biofilm formation of strains was tested by microtiter dish biofilm assay. (**D**) Strains were cultured in BM with different concentrations of exogenous CSP. Biofilm formation ability was tested by microtiter dish biofilm assay. Means and standard deviations from triplicate experiments are shown. ND: no significant difference, *P ≤ 0.05, **P ≤ 0.001, ND: no significant difference, Student’s *t-*test.

Previous studies have demonstrated that *ciaR* is a genetic competence inhibitor in *Streptococcus pneumoniae* ^28–30^ and *Streptococcus mutans* ^48^. In our RNA sequencing data, most of the *com* genes (*comC*, *comD*, *comE*, *comEA*, *comEC*, *comFA*, *comFC*, *comX*, *comYA-D* and*comGF*) were up-regulated (Table 1) while *htrA* was down-regulated in Δ*ciaR* (Supplementary Dataset). The data above suggest that *ciaR* is a repressor of the genetic competence system in *S*. *sanguinis*. Due to the up-regulation of *comC* transcript levels in Δ*ciaR*, we expected that Δ*ciaR* produced more CSP than WT because the *comC* gene encodes the CSP precursor in *S*. *sanguinis* ^49,50^. To compare the concentration of CSP in WT and Δ*ciaR*, we measured the transformation efficiency of Δ*comC* supplemented with either the supernatant or cell lysate of WT and Δ*ciaR*. Compared with WT, the addition of the supernatant or cell lysate from Δ*ciaR* elevated the transformation efficiency of Δ*comC* to a greater extent, suggesting a higher concentration of CSP produced by the *ciaR* mutant (Fig. 6A and B).

**Table 1:** Transcript levels of genetic competence-related genes in Δ*ciaR* and Δ*ciaR*/*ciaR*.

Synonym	Name	Annotation	Δ*ciaR*		Δ*ciaR*/*ciaR*	
			fold change	qvalue	fold change	qvalue
SSA_2394	*comC*	competence stimulating peptide	24.46	0.00E + 00	0.84	1.00E + 00
SSA_2379	*comD*	signal transduction protein	35.15	0.00E + 00	0.93	7.73E−01
SSA_2378	*comE*	two-component system LytR/AlgR family transcriptional regulator	12.76	0.00E + 00	0.74	8.54E−01
SSA_0715	*comEA*	DNA uptake protein	93.00	0.00E + 00	1.00	1.00E + 00
SSA_1497	*comEB*	dCMP deaminase	1.24	5.07E−06	1.07	5.70E−02
SSA_0716	*comEC*	competence protein	78.00	0.00E + 00	1.00	1.00E + 00
SSA_1836	*comFA*	superfamily II ATP-dependent DNA/RNA helicase	78.00	0.00E + 00	1.00	1.00E + 00
SSA_1835	*comFC*	late competence protein	94.00	0.00E + 00	1.00	1.00E + 00
SSA_0189	*comGF*	competence protein ComGF	81.00	0.00E + 00	1.00	1.00E + 00
SSA_0016	*comX*	ComX1, transcriptional regulator of competence-specific genes	56.25	0.00E + 00	0.51	1.20E−02
SSA_0184	*comYA*	competence protein ComYA	226.00	0.00E + 00	1.00	1.00E + 00
SSA_0185	*comYB*	competence protein ComYB	143.00	0.00E + 00	1.00	1.00E + 00
SSA_0186	*comYC*	competence protein ComYC	62.00	0.00E + 00	1.00	1.00E + 00
SSA_0187	*comYD*	competence protein ComYD	76.00	0.00E + 00	1.00	1.00E + 00
SSA_2246	*cinA*	competence damage-inducible protein A	11.05	0.00E + 00	1.00	2.84E−01
SSA_0749	*coiA*	competence protein	8.00	0.00E + 00	1.00	1.00E + 00

### The over-expression of genetic competence genes in Δ*ciaR* does not lead to the fragile biofilm phenotype

Three experiments were done to explore whether Δ*ciaR* was able to alter biofilm formation by producing more CSP. First, a Δ*ciaR*Δ*comC* double mutant was constructed. The addition of the *comC* mutation did not affect the biofilm formation ability of Δ*ciaR* (Fig. 6C). Second, although a very high concentration of exogenous CSP (10 μg/mL) could slightly decrease biofilm biomass at the early stage of biofilm formation (8 hours, data not shown), in our experimental conditions, the addition of exogenous CSP did not affect biofilm biomass (Fig. 6D). Third, three *com* gene mutants, Δ*comC*, Δ*comD* and Δ*comE*, all of which eliminate competence, were unaffected for biofilm formation (Fig. 6E). Taken together, the results indicate that *ciaR* does not regulate biofilm formation by repressing the expression of genetic competence genes.

## Discussion

Our results suggest that the Δ*ciaR* mutation disrupts normal biofilm formation in *S*. *sanguinis* predominantly if not entirely by increasing expression of the arginine biosynthetic pathway resulting in increased arginine levels, which reduces transcription of the *gtfP* gene encoding glucosyltransferase, which reduces production of water-insoluble glucan (WIG)—the major component of *S*. *sanguinis* biofilms. (In experiments not shown, soluble glucan was produced at levels nearly 50 times lower than WIG, confirming that WIG is the predominant glucan in *S*. *sanguinis* biofilms.) There are data supporting each part of this model. The Δ*ciaR* mutation significantly reduces *gtfP* expression and significantly increases *arg* gene expression (Figs 3 and 4). Exogenous arginine also reduces *gtfP* expression, biofilm formation, and WIG production (Figs 4 and S5). Exogenous ornithine, an arginine precursor, also reduces biofilm formation, but not in a mutant incapable of converting ornithine to arginine (Fig. S7). The Δ*ciaR* strain produces significantly less biofilm in the standard assay than its wild-type parent, while most of the *arg* mutants produce more, and all of the Δ*ciaR arg* double mutants produce WT levels of biofilm (Figs 1, 4 and S3), consistent with the model of *ciaR* affecting biofilm formation through its effect on *arg* gene expression. Nearly identical results are observed if WIG production is measured rather than biofilm formation (Figs 4 and S2). The *arg* mutants do not show increased biofilm formation in the absence of sucrose, which is the substrate for GtfP (Fig. S6). We also ruled out some alternative explanations for our results. In multiple experiments, we found no support for either the Δ*ciaR* mutation or the *arg* mutations affecting biofilm formation through effects on growth (Figs 2, S2 and S4). We also ruled out the possibility that the Δ*ciaR* mutation affected biofilm formation through its effect on competence induction, which could otherwise have been a reasonable explanation (Fig. 6). Related to this final result, Rodriguez *et al*. added CSP to a Δ*comC* mutant of *S*. *sanguinis* SK36 and tested gene expression by microarray. In their study, genes responsible for arginine biosynthesis were not regulated by the addition of CSP^50^. Thus, *ciaR* appears to control genetic competence and arginine biosynthesis independently.

In one sense, the effect of arginine on biofilm formation should not have been that surprising, in that there have been many studies showing the effect of exogenous L-arginine on polysaccharide production and biofilm formation in *S*. *pneumoniae* and *S*. *mutans*. In those studies, however, the mechanism by which this occurred was not determined^16–18,51,52^. Further, we are unaware of any previous studies that have examined the relationship between arginine biosynthesis and biofilm formation.

One remaining question concerns the Δ*argB* mutant. Although this mutant was like most of the other *arg* biosynthetic mutants in many respects, including demonstrating increased expression in Δ*ciaR* (Fig. 3B,C), increased WIG production (Figs 4 and S5), and increased biofilm formation in sucrose compared to glucose (Fig. S6), the Δ*argB* strain was unique in that it severely aggregated in both the planktonic (Fig. S4) and biofilm (Fig. 1C) states. This reason for this difference is not clear, although one possibility is that accumulation of N-acetyl-glutamate, the substrate of the ArgB enzyme (acetylglutamate kinase), is responsible for this unique phenotype. Further studies will be required to answer this question.

Another question concerns the mechanism by which CiaR controls *arg* gene expression. Previous studies have identified genes directly regulated by CiaR in *S*. *pneumoniae* and *Bacillus anthracis* ^28,53^. To predict genes directly regulated by CiaR in *S*. *sanguinis*, we collected 200 bp of sequence upstream of each ORF to establish a promoter database for *S*. *sanguinis* SK36. Three CiaR binding sequences reported in *S*. *pneumoniae* (NTTNAG-N5-TTTAAN, NTTNAG-N5-TTTTAN and NTTNAG-N5-ATTAAN) were searched against the database and the results are shown in Table S2 ^28^. There were 17 genes predicted to have a CiaR binding sequence, including *htrA* and *comE* (Table S2). The expression of most of these genes was significantly changed in Δ*ciaR* (Table S2). In addition, previous studies have shown that CiaR directly binds to the promoter of *htrA* and controls its expression in *S*. *pneumoniae*, which suggests that CiaR of *S*. *sanguinis* may have a similar binding site to that of *S*. *pneumoniae* ^28^. The arginine biosynthetic genes are distributed among three different operons in *S*. *sanguinis* SK36 (Fig. S9). None of these three operons was predicted to contain a CiaR binding site, which suggests that the arginine biosynthetic genes are regulated by *ciaR* indirectly (Table S2). Some other transcriptional regulators like *mga* listed in Table S2 were also controlled by *ciaR*, which might explain the effect of *ciaR* on *arg* gene transcription. The mechanism by which *ciaR* regulates the expression of *arg* genes will require further study.

## Materials and Methods

### Bacterial strains, growth and antibiotics

Strains and plasmids used in this study are listed in Table S3. Unless otherwise stated, strains were grown in brain heart infusion broth (BHI; Difco Inc., Detroit, MI) media overnight and then diluted 100-fold into biofilm media (BM) and incubated under microaerobic conditions (6% O_2_, 7.2% CO_2_, 7.2% H_2_ and 79.6% N_2_) at 37 °C C using an Anoxomat^®^ system (Spiral Biotech, Norwood, MA). BM was supplemented with 1% sucrose for the growth of static biofilms and the measurement of bacterial growth^54^. Kanamycin was added to a concentration of 500 μg/ml for mutant cultures.

### Mutant construction and complementation

For double mutant construction, three sets of primers were used to independently PCR amplify the 1-kb sequence of the upstream fragment of *ciaR*, the downstream fragment of *ciaR* and the *erm* gene for erythromycin resistance. The three fragments were combined through a second round of PCR. The final recombinant PCR product was transformed into *S*. *sanguinis* SK36 single mutants. Double mutants were selected by erythromycin resistance and confirmed by PCR analysis. For complementation of the *ciaR* mutant, a similar PCR-based method was employed^55^. Briefly, three DNA fragments were independently amplified, the 1-kb sequence upstream plus the coding sequence of *ciaR*, the erythromycin resistance cassette (from the plasmid pVA838) and the 1-kb sequence downstream of *ciaR*. Overlapping PCR was done to generate the final recombinant PCR product. It was then introduced into Δ*ciaR* to replace the kanamycin resistance cassette with the *ciaR* ORF and the erythromycin resistance cassette. An erythromycin resistant and kanamycin sensitive transformant was selected and confirmed by PCR analysis.

### Microtiter dish biofilm assay

Overnight cultures were diluted 1:100 into BM in a 96-well microtiter plate (Falcon 3911). After incubation at 37 °C for 24 hours under microaerobic conditions, the planktonic cells were removed and biofilms were washed once with distilled water, and stained by the addition of 0.4% crystal violet (CV) for 30 minutes at room temperature. CV was then removed with a pipette and biofilms were washed twice with distilled water, solubilized in 30% acetic acid and measured at A_560_ as described previously^42^. Three replicates were examined and data were analyzed by Student’s *t-*test.

### Static biofilm assay

Static biofilms were grown in 4-chambered glass coverslip wells (Chambered #1.5 German Coverglass System, Nunc) in BM at 37 °C under micro-aerobic conditions for 24 hours. The supernatant was discarded, and biofilms were washed by PBS and stained stained with a live/dead staining kit (Molecular Probes, Invitrogen) in darkness for 10 minutes. The fluorescent and differential interference microscopy (DIC) images were acquired with a Zeiss LSM710 confocal laser scanning microscope (Zeiss, Germany) and quantified by COMSTAT in Matlab software^38^. Three images of each sample were quantified to calculate the means and standard deviations. The signal of SYTO9 (green) showed live cells; the signal of PI (red) was dead cells and eDNA; the biomass / average thickness ratio represented cell density inside of biofilms.

### Biofilms in a flow cell system

Two fluorescent proteins, mTFP1 (green) and mCherry (red), were expressed from the plasmids pVMTeal and pVMcherry, respectively^47^. BM with 10 μg/mL erythromycin was pre-incubated in a jar under microaerobic conditions 3 days prior to the experiment. Strains were cultured in BHI under microaerobic conditions overnight and then pumped into the channels of a microfluidic chip (straight channel chips, productor cord: 01-0176-0142-01, ChipShop) with a flow speed of 0.1 mL/minute for 1 minute by using an NE-1200 Twelve Channel Programmable Syringe Pump (Newera, USA). Flow was stopped for 30 minutes to allow cells to attach. The syringes with bacterial cultures were then discarded and new syringes with prepared BM were linked with the chip. The pump was turned on again with a speed of 0.1 mL/minute for 1 minute to wash away unattached cells and remaining BHI. The flow speed was then changed to 0.1 μL/minute for biofilm formation. Images were obtained with a Zeiss Axiovert 200 M fluorescent microscope (Zeiss, German) at different time points. Four days later, biofilms in the chip were observed with a Zeiss LSM710 confocal laser scanning microscope. The images were analyzed by COMSTAT^38^. The strength of the fluorescence signal represented biofilm biomass. Ten images from each sample were quantified to calculate the means and standard deviations.

### Growth curve measurement

Strains were cultured in 96-well plates with continuous shaking, and growth was monitored every 15 minutes at 600 nm with a Synergy H1 Hybrid Reader (BioTek, USA). The microaerobic condition (6% O_2_, 6% CO_2_) was maintained by injection of CO_2_ and N_2_ to maintain CO_2_/O_2_ set concentrations (BioTek, USA). Three replicates were examined to calculate the means and standard deviations.

### RNA sequencing

WT and Δ*ciaR* were cultured in TSB medium supplemented with 1% (w/v) sucrose overnight and then diluted into fresh TSB supplemented with 1% sucrose and grown for 3 hours in anaerobic conditions at 37 °C. Samples were collected, treated with RNA protect bacteria reagent (Qiagen, Valencia, CA) for 5 min to stabilize RNA and stored at −80 °C. Cells were lysed by mechanical disruption using FastPrep lysing matrix B (Qbiogene, Irvine, CA). Total RNA was treated with DNase I (Qiagen) and prepared using RNA easy mini kits (Qiagen) according to the manufacturer’s instructions. Ribo-Zero Magnetic Kit for Bacteria (Illumina) was used to deplete ribosomal RNA from 2 µg of total RNA. NEBNext Ultra Directional RNA Library Prep Kit for Illumina (New England BioLabs) was used for the following RNA sequencing library preparation according to the manufacturer’s protocol. Library sequencing was performed by the Nucleic Acids Research Facilities at Virginia Commonwealth University using an Illumina HiSeq. 2000 instrumen. The raw RNA sequencing data are available in the NCBI Gene Expression Omnibus (GEO) (www.ncbi.nlm.gov/geo/query) under the accession number: GSE99864. Reads obtained from RNA sequencing were aligned against the *S*. *sanguinis* SK36 genome using Rockhopper v. 2.03^56^. Analyses were run using default parameter settings. Significance was determined by a q-value adjusted for a false discovery rate of 1%. Transcriptome profiles were analyzed for enriched pathways and functionally related genes using DAVID v. 6.8 Beta^57,58^. Four replicates were examined for data analysis.

### Competence assay

Competence of *S*. *sanguinis* strains was determined by transformation with pJFP96, a suicide plasmid containing the spectinomycin resistance gene (*aad*9) with ~1 kb upstream and downstream of the SSA_0169 as described previously^59^. Briefly, overnight cultures were diluted 200-fold into Todd Hewitt broth containing 2.5% horse serum (Fisher scientific, Pittsburgh, PA) and incubated microaerobically for 3 hours. Culture aliquots of 300 μL were transferred into pre-warmed microfuge tubes containing 50 ng of pJFP96 and incubated for 1 h at 37 °C. Cells were serially diluted, plated on BHI agar plates with and without spectinomycin (100 μg/ml) and grown microaerobically for 2 days at 37 °C. The transformation efficiency was defined as the ratio of spectinomycin-resistant colonies to total CFU. Three replicates were analyzed to calculate the means and standard deviations.

### High-throughput biofilm assay

Biofilms were tested by a protocol similar to the microtiter dish biofilm assay. The differences were: OD_600_ was tested before CV staining; CV and water were injected into 96 wells at the CV staining step and washing step respectively by using a Caliper Sciclone G3 liquid handling robot (PerkinElmer, USA) with an injection speed of 100 μL/second. All of the mutants used in the experiment were generated by Ge *et al*.^37^.

### Polysaccharide production assay

Cells were cultured in BM under microaerobic conditions at 37 °C for 24 hours at 200 rpm, harvested by centrifugation and then diluted to a density of 10^6^/mL. Extracellular polysaccharide was stained by HHA-FITC (EY Labs, USA) as previously described^22^. HHA-FITC was used at a final concentration of 100–200 mg/mL for 1 hour in darkness and fluorescence signal was measured using a Guava® EasyCyte Flow Cytometer.

WIG was measured as previously described^15^. Biofilms were grown in BM for 24 hours in 24-well plates. The supernatant was then removed and biofilms were resuspended in 1 mL of distilled water. One-half mL of cell suspension was prepared for the determination of total protein concentration. Another 500 μL of bacterial suspension was centrifuged. The sediment was dissolved in the same volume of 1 N NaOH for 3 hours, and centrifuged. The supernatants were precipitated by 3 volumes of isopropanol overnight at −20 °C. The precipitates obtained by centrifugation were then air dried, and dissolved in 100 μL of 1 N NaOH. The amount of glucans in each fraction was quantified by the phenol-sulfuric acid method as previously described^60^. Glucose was used as a reference carbohydrate to generate a standard curve. The concentration of WIG was normalized by total protein concentration in the biofilm. Four replicates were examined to calculate the means and standard deviations.

### qRT-PCR assay

RNA extraction was performed as described above for the RNA sequencing assay. Reverse transcription followed the standard procedure provided with the SuperScript™ III Reverse Transcriptase Kit (Qiagen). The cDNA was used as the template, combined with 2X SYBR Green PCR Master Mix (Qiagen) and the q-PCR primers showed in Table S4. Gene expression in Δ*ciaR* is relative to that in WT. Three replicates were analyzed to calculate the means and standard deviations.

### The measurement of protein concentration

Cells were harvested and resuspended in lysis buffer (Tris pH 7.4 50 mM, NaCl 150 mM, glycerol 10%, NP-40 1%, SDS 0.1%). Cell suspensions were incubated on ice for 30 min and then lysed by mechanical disruption using FastPrep lysing matrix B (Qbiogene, Irvine, CA). The protein concentration of cell lysate was measured by following the standard protocol of Pierce^TM^ BCA Protein Assay Kit (Thermo Scientific). Four replicates were analyzed to calculate the means and standard deviations.

### DAVID gene function classification

All the genes whose expression was significantly altered in Δ*ciaR* were input into DAVID database (https://david.ncifcrf.gov/summary.jsp)^61^. The KEGG_pathway option was chosen for functional annotation clustering.

### Statistical analysis

All data were obtained from at least three biological replicates. Student’s t-test was applied to analyze data on CFU, biofilm assay, qRT-PCR, WIG and competence assay. The data of biofilm formation in Fig. 4A were analyzed by two-way ANOVA. In flow cell experiments, ten images from fluorescence microscopy were analyzed to calculate the means and standard deviations. The RNA sequencing data were analyzed by Rockhopper software^62^.

### Data Availability

The datasets generated during and/or analyzed during the current study are available from the corresponding author upon reasonable request.

#### Accession code

GSE99864.

## Electronic supplementary material

Supporting information

Supporting dataset
