# Supplementary material for: ciaR impacts biofilm formation by regulating an arginine biosynthesis pathway in Streptococcus sanguinis SK36
Source: Sci Rep. 2017 Dec 7;7:17183. doi: 10.1038/s41598-017-17383-1 (PMC5719415; doi:10.1038/s41598-017-17383-1)
Supplement: Supplementary file 1 — Supporting information [file 41598_2017_17383_MOESM1_ESM.pdf]

ciaR impacts biofilm formation by regulating an arginine biosynthesis pathway in *Streptococcus sanguinis* SK36

Bin Zhu[1], Xiuchun Ge[1], Victoria Stone[1], Xiangzhen Kong[1], Fadi Elrami[1], Yan Liu[1,2], Todd Kitten[1,3] and Ping Xu[1,3,*]

Supporting information

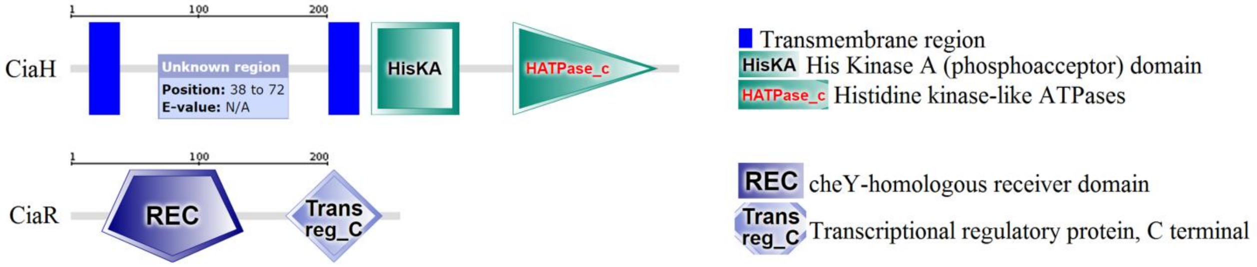

Fig. S1 The secondary structures of CiaH and CiaR predicted by SMART.

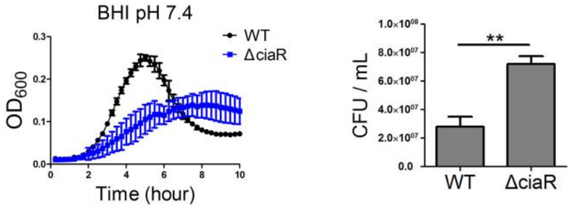

Fig. S2 Growth of WT and Δ*ciaR* in BHI. WT and Δ*ciaR* were cultured in BHI with continuous shaking. OD_{600} was monitored with a Synergy H1 Hybrid Reader every 15 minutes (left) and CFUs were determined after 10 hours of incubation (right). Means and standard deviations from triplicate experiments are shown. **$P \leq 0.01$, Student's *t*-test.

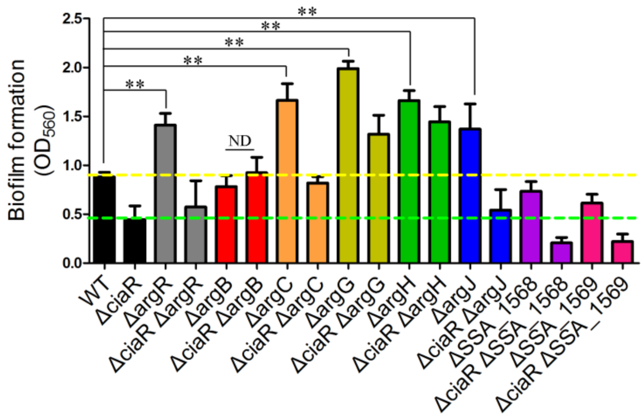

Fig. S3 Biofilm biomass of strains was examined by microtiter dish biofilm assay. Means and standard deviations from triplicate experiments are shown. **P ≤ 0.01, ND: no significant difference, Student's t-test.

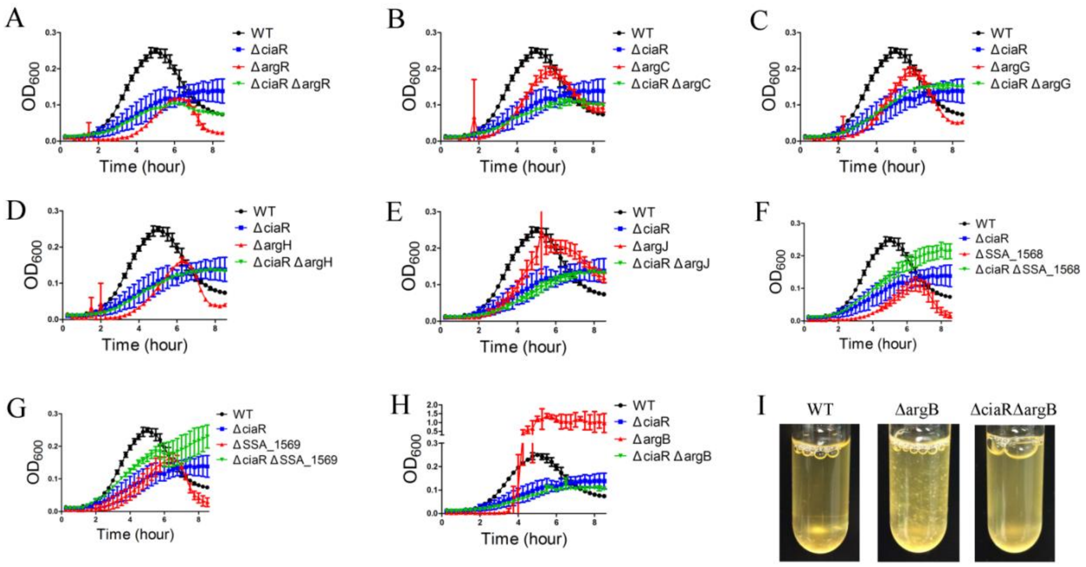

Fig. S4 The growth of arginine biosynthesis-related gene mutants. (A-H) The growth curves of strains in BHI medium grown with continuous shaking. (I) Aggregation of the indicated strains in BHI media.

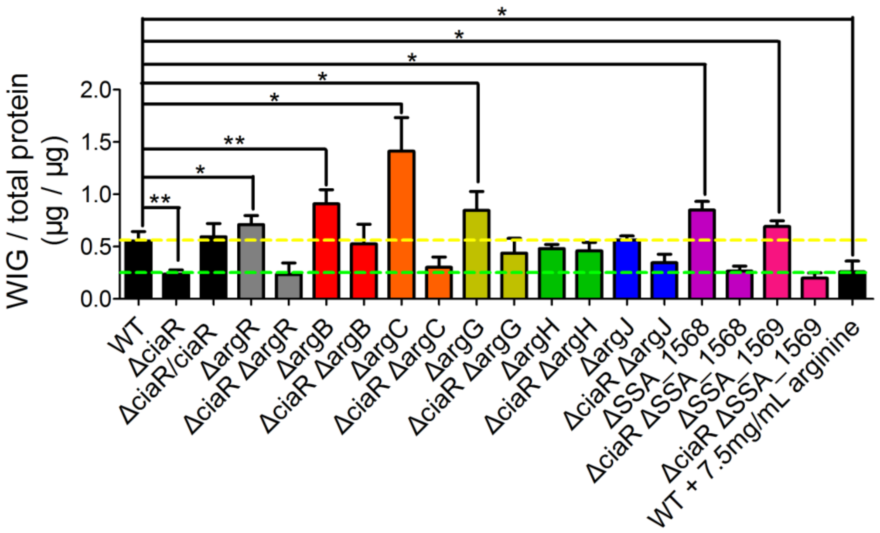

Fig. S5 The concentration of WIG in the biofilms of selected strains. *P ≤ 0.05, **P ≤ 0.01, Student's *t*-test. Means and standard deviations from triplicate experiments are shown.

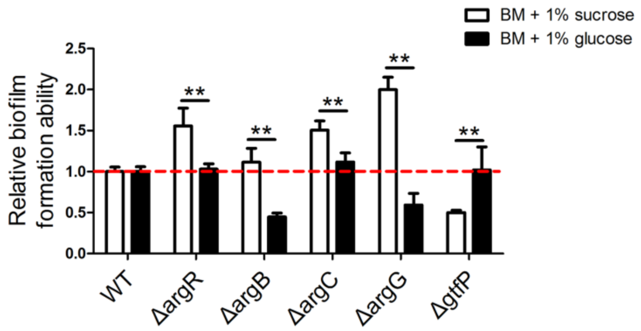

Fig. S6 Relative biofilm formation ability of *arg* mutants in BM + 1% glucose or sucrose. Δ*argR*, Δ*argB*, Δ*argC*, Δ*argG*, Δ*argH*, Δ*argJ* and Δ*gtfP* cultured in BM + 1% glucose or BM + 1% sucrose for 24 hours. Relative biofilm formation ability was measured by microtiter dish biofilm assay. **P ≤ 0.01, Student's *t*-test. Means and standard deviations from triplicate experiments are shown.

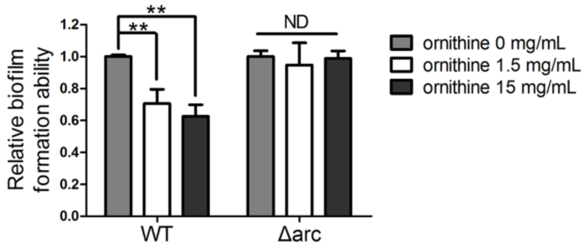

Fig. S7 The effect of L-ornithine on biofilm formation. WT and Δ*arc* (SSA_0738) were cultured in BM with different concentrations of L-ornithine. Biofilm formation ability was tested by microtiter dish biofilm assay. Means and standard deviations from four experiments are shown. **P ≤ 0.01, ND: no significant difference, Student's *t*-test.

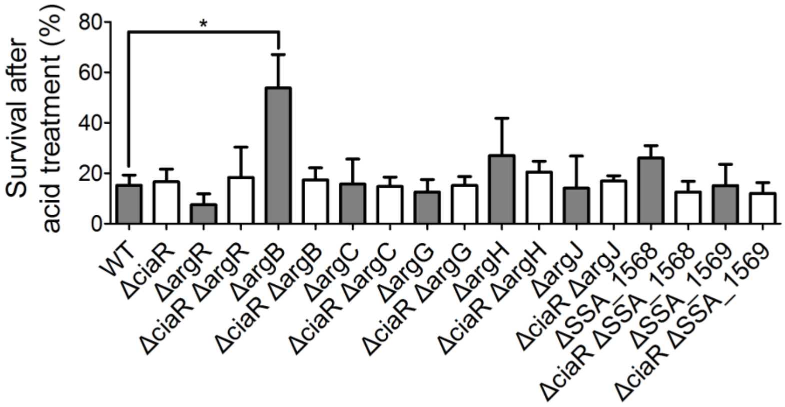

Fig. S8 Tolerance of strains to acid treatment. The tolerance of strains to acid conditions was tested by treatment of cells with an acetic acid - sodium acetate buffer at pH 4.8 for 30 minutes. The CFUs before and after acid treatment were examined to define the survival ratio. *P ≤ 0.05, Student's *t*-test. Means and standard deviations from triplicate experiments are shown.

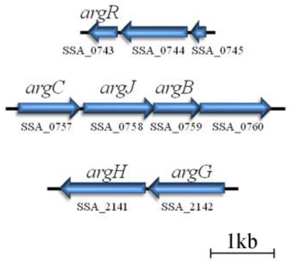

Fig. S9 The location of *arg* genes in the genome of *S. sanguinis*.

Table S1 Two-way ANOVA to analyze the impact of gene mutations on biofilm formation

	Interaction	P value	Column Factor	P value	Row Factor	P value
ΔargR (Row) VS. ΔciaR (column)	3.81	0.0043	77.47	< 0.0001	15.00	< 0.0001
ΔargB (Row) VS. ΔciaR (column)	16.73	0.0003	50.17	< 0.0001	25.32	< 0.0001
ΔargC (Row) VS. ΔciaR (column)	15.97	< 0.0001	48.61	< 0.0001	33.89	< 0.0001
ΔargG (Row) VS. ΔciaR (column)	8.53	< 0.0001	44.58	< 0.0001	46.16	< 0.0001
ΔargH (Row) VS. ΔciaR (column)	0.04	0.6056	43.41	< 0.0001	54.83	< 0.0001
ΔargJ (Row) VS. ΔciaR (column)	2.92	0.0545	72.47	< 0.0001	16.90	0.0002
ΔSSA_1568 (Row) VS. ΔciaR (column)	1.94	0.0515	84.97	< 0.0001	9.16	0.0002
ΔSSA_1569 (Row) VS. ΔciaR (column)	0.02	0.8258	95.03	< 0.0001	0.59	0.2254

Table S2 The prediction of CiaR binding genes in *S. sanguinis* SK36

Synonym	Name	Product	fold change in Δ*ciaR*	qValue	predicted binding sequence	searched sequence
SSA_0316	-	hypothetical protein	1.313	5.74E-06	TTTTAGCCAGATTTTAG	NTTNAG-N5-TTTTAN
SSA_0674	*xseA*	exodeoxyribonuclease VII large subunit	1.357	7.50E-06	TTTCAGGGGATTTTTAG	NTTNAG-N5-TTTTAN
SSA_0701	-	cation transporter	1.361	1.85E-05	TTTTAGGGAAATTTTAA	NTTNAG-N5-TTTTAN
SSA_0753	*prsA*	foldase protein PrsA	0.845	8.22E-01	TTTTAGTAAAATTTAAG	NTTNAG-N5-TTTAAN
SSA_0958	-	hypothetical protein	0.176	1.44E-16	TTTCAGCCTTTTTTAAG	NTTNAG-N5-TTTAAN
SSA_1070	-	ribosomal large subunit pseudouridine synthase D	1.365	1.89E-05	TTTCAGTTAGATTTTAT	NTTNAG-N5-TTTTAN
SSA_1378	-	hypothetical protein	1.320	1.19E-05	ATTTAGAGTCTTTTTAA	NTTNAG-N5-TTTTAN
SSA_1424	-	hydrolase	1.455	1.37E-08	ATTAAGATAAGTTTTAA	NTTNAG-N5-TTTTAN
SSA_1624	-	hypothetical protein	1.133	6.76E-04	TTTTAGCCATTTTTAAA	NTTNAG-N5-TTTAAN
SSA_1928	-	acyl-CoA dehydrogenase	1.318	1.94E-03	GTTAAGACCTAATTAAC	NTTNAG-N5-ATTAAN
SSA_2151	*mga*	M protein trans-acting positive transcriptional regulator	0.300	4.77E-09	TTTTAGATATATTTTAG	NTTNAG-N5-TTTTAN
SSA_2192	-	hypothetical protein	6.768	2.75E-274	TTTAAGAATTTTTTAAA	NTTNAG-N5-TTTAAN
SSA_2251	-	hypothetical protein	0.310	1.31E-05	TTTTAGTAACATTTTAA	NTTNAG-N5-TTTTAN
SSA_2312	-	hypothetical protein	7.889	3.11E-267	TTTAAGGGTTCATTAAC	NTTNAG-N5-ATTAAN
SSA_2378	*comE*	two-component system LytR/AlgR family transcriptional regulator	12.762	0.00E+00	TTTTAGACACATTTTAA	NTTNAG-N5-TTTTAN
SSA_2381	*htrA*	DegP protein	0.240	9.41E-10	TTTAAGTTATAATTAAG	NTTNAG-N5-ATTAAN
SSA_2382	-	chromosome partitioning protein ParB	0.262	7.77E-08	GTTAAGCTCAAATTAAC	NTTNAG-N5-ATTAAN

Table S3 Strains and plasmids used in this study.

Strain or plasmid	Genotype and/or relevant characteristics	Source or reference
S. sanguinis strains		
SK36	Wild type, Human plaque isolate	[1]
Δ*ciaR*	SK36, insert-deletion of *ciaR* gene, Km^r	[2]
Δ*ciaR*/*ciaR*	*km* gene in Δ*ciaR*was replaced by the insertion of *ciaR*-*erm*, Erm^r	This study
Δ*comC*	SK36, insert-deletion of *comC* gene, Km^r	[2]
Δ*comD*	SK36, insert-deletion of *comD* gene, Km^r	[2]
Δ*comE*	SK36, insert-deletion of *comE* gene, Km^r	[2]
Δ*argR*	SK36, insert-deletion of *argR* gene, Km^r	[2]
Δ*argB*	SK36, insert-deletion of *argB* gene, Km^r	[2]
Δ*argC*	SK36, insert-deletion of *argC* gene, Km^r	[2]
Δ*argG*	SK36, insert-deletion of *argG* gene, Km^r	[2]
Δ*argH*	SK36, insert-deletion of *argH* gene, Km^r	[2]
Δ*argJ*	SK36, insert-deletion of *argJ* gene, Km^r	[2]
ΔSSA_1568	SK36, insert-deletion of SSA_1568, Km^r	[2]
ΔSSA_1569	SK36, insert-deletion of SSA_1569, Km^r	[2]
Δ*ciaR* Δ*comC*	SK36, insert-deletion of *ciaR* gene based on Δ*comC*, Km^r Erm^r	This study
Δ*ciaR* Δ*argR*	SK36, insert-deletion of *ciaR* gene based on Δ*argR*, Km^r Erm^r	This study
Δ*ciaR* Δ*argB*	SK36, insert-deletion of *ciaR* gene based on Δ*argB*, Km^r Erm^r	This study
Δ*ciaR* Δ*argC*	SK36, insert-deletion of *ciaR* gene based on Δ*argC*, Km^r Erm^r	This study
Δ*ciaR* Δ*argG*	SK36, insert-deletion of *ciaR* gene based on Δ*argG*, Km^r Erm^r	This study
Δ*ciaR* Δ*argH*	SK36, insert-deletion of *ciaR* gene based on Δ*argH*, Km^r Erm^r	This study
Δ*ciaR* Δ*argJ*	SK36, insert-deletion of *ciaR* gene based on Δ*argJ*, Km^r Erm^r	This study
Δ*ciaR* ΔSSA_1568	SK36, insert-deletion of *ciaR* gene based on ΔSSA_1568, Km^r Erm^r	This study
Δ*ciaR* ΔSSA_1569	SK36, insert-deletion of *ciaR* gene based on ΔSSA_1569, Km^r Erm^r	This study
Plasmids		
pVMTeal	Gram-positive replicative plasmid carrying *S. mutansldh* promoter and codon-optimized gene for mTFP1; erm^R	[3]
pVMcherry	Gram-positive replicative plasmid carrying *S. mutansldh* promoter and codon-optimized gene for mCherry; erm^R	[3]

Table S4 primers used in qRT-PCR experiments

name	Sequence (5'-3')	Application
F-qpcr-gtfP	GCCCAAATTCTCAACCGTTAC	q-PCR of *gtfP* gene
R-qpcr-gtfP	ATCTTGCCCTTGACTTGGTAG	q-PCR of *gtfP* gene
F-qpcr-gyrA	AGCTGATTGCCTTGATTGCAGAC	q-PCR of *gyrA* gene
R-qpcr-gyrA	ATCCGCAAATTTACGCTTGACCT	q-PCR of *gyrA* gene
F-qpcr-argR	GCGTCATTGTAACCCAGTCC	q-PCR of *argR* gene
R-qpcr-argR	GGATGGTGCTATGCTATTGATG	q-PCR of *argR* gene
F-qpcr-argC	GCCTTGATTCCCTTGCTG	q-PCR of *argC* gene
R-qpcr-argC	TGGATGCGGCTGGATT	q-PCR of *argC* gene
F-qpcr-argJ	GCTATCGGCTATGCGGG	q-PCR of *argJ* gene
R-qpcr-argJ	CATCACTTGTTTCCTCGGG	q-PCR of *argJ* gene
F-qpcr-argB	CAGGTGTCATCAAGGGTGGTA	q-PCR of *argB* gene
R-qpcr-argB	CTATCAAGGTGCCCGTCAA	q-PCR of *argB* gene
F-qpcr-argH	TGAGTTTGATGTGCGTAATGAA	q-PCR of *argH* gene
R-qpcr-argH	GGAGCGAGCCGTATGAAG	q-PCR of *argH* gene
F-qpcr-argG	GGTGTTGGGCGGATTG	q-PCR of *argG* gene
R-qpcr-argG	CGATTTCCTTGTGGGCAG	q-PCR of *argG* gene
F-qpcr-1568	TTGGACTGCGGTTCTCTGT	q-PCR of SSA_1568 gene
R-qpcr-1568	GATAGTGCGGCTTCGGTC	q-PCR of SSA_1568 gene
F-qpcr-1569	CTTTACTTCTGCTGCTTTGGC	q-PCR of SSA_1569 gene
R-qpcr-1569	CGAATAGCCTGAGGTAGGATGA	q-PCR of SSA_1569 gene

1 Kilian, M. & Holmgren, K. Ecology and nature of immunoglobulin A1 protease-producing streptococci in the human oral cavity and pharynx. *Infection and immunity* **31**, 868-873 (1981).

2 Ge, X. & Xu, P. Genome-wide gene deletions in Streptococcus sanguinis by high throughput PCR. *Journal of visualized experiments : JoVE*, doi:10.3791/4356 (2012).

3 Vickerman, M. M., Mansfield, J. M., Zhu, M., Walters, K. S. & Banas, J. A. Codon-optimized fluorescent mTFP and mCherry for microscopic visualization and genetic counterselection of streptococci and enterococci. *Journal of microbiological methods* **116**, 15-22, doi:10.1016/j.mimet.2015.06.010 (2015).
